# Concentrated Exposure Therapy vs Internet-Based Cognitive Behavioral Therapy for Obsessive-Compulsive Disorder: A Randomized Clinical Trial

**DOI:** 10.1001/jamanetworkopen.2026.39281

**Published:** 2026-10-19

**Authors:** Lena Jelinek, Lara Wille, Jakob Scheunemann, Luisa Tegtmeier, Holger Zapf, Franziska Miegel, Lea Schuurmans, Josephine Schultz, Jürgen Gallinat, Amir H. Yassari

**Affiliations:** 1 Department of Psychiatry and Psychotherapy, University Medical Center Hamburg-Eppendorf, Hamburg, Germany; 2 Department of Child and Adolescent Psychiatry, Psychotherapy and Psychosomatics, University Medical Center Hamburg-Eppendorf, Hamburg, Germany

## Abstract

**Importance:**

Given the high prevalence, chronicity, and associated disability of obsessive-compulsive disorder (OCD), optimizing treatment is a global priority. The Bergen 4-day treatment (B4DT), a concentrated exposure-based intervention, may reduce treatment burden and improve access to evidence-based care.

**Objective:**

To evaluate the efficacy of B4DT compared with internet-based cognitive behavioral therapy (iCBT) in reducing OCD symptom severity.

**Design, Setting, and Participants:**

Single-center randomized controlled trial (1:1 allocation). Between May 8, 2024, and August 8, 2025, adults (aged 18 to 72 years) with OCD were enrolled and were followed up for 3 months. Key exclusion criteria included schizophrenia, bipolar disorder, acute suicidality, substance dependence, and recent psychopharmacological changes. Outcomes were assessed at baseline, 6 weeks, 8 weeks (posttreatment), and 3 months (follow-up) by masked evaluators.

**Interventions:**

Patients were randomized to B4DT (including preparatory sessions) or an 8-week unguided iCBT program.

**Main Outcomes and Measures:**

The primary outcome was the change in OCD symptom severity, measured by the Yale-Brown Obsessive Compulsive Scale (Y-BOCS). Secondary outcomes included self-reported OCD (OCI-R), dysfunctional cognitions, quality of life, depressive symptoms, experiential avoidance, global functioning, self-esteem, and treatment satisfaction.

**Results:**

A total of 253 individuals were screened, and 119 were enrolled after 53 were found to be ineligible and 81 declined to participate. Sixty of the 119 randomized participants (mean [SD] age, 33.5 [10.1] years; 79 [66.4%] women) were assigned to B4DT and 59 to iCBT. The primary analysis included all randomized participants (intention-to-treat). B4DT was associated with significantly greater reductions in OCD symptoms (Y-BOCS) than iCBT, with large effects at posttreatment (mean [SE] difference, −7.9 [0.9]; 95% CI, −9.7 to −6.0; Hedges *g*, −1.6) and sustained effects at 3-month follow-up (mean [SE] difference, −5.6 [1.1]; 95% CI, −7.8 to −3.4; Hedges *g*, −1.0). Secondary outcomes consistently favored B4DT. Response and remission rates at 3-month follow-up were higher with B4DT (35 of 57 [61.4%] vs 11 of 44 [25.0%] and 24 of 57 [42.1%] vs 6 of 44 [13.6%], respectively).

**Conclusions and Relevance:**

In this randomized clinical trial of 119 adults with OCD, B4DT was superior to iCBT in reducing OCD symptom severity, with sustained benefits. Its brief, concentrated format may represent a scalable strategy to improve access to effective OCD treatment globally.

**Trial Registration:**

German Clinical Trials Register: DRKS00034236

## Key Points

**Question:**

Is the Bergen 4-day treatment for obsessive-compulsive disorder superior to internet-based cognitive behavioral therapy in reducing obsessive-compulsive symptoms?

**Findings:**

In this randomized clinical trial of 119 adults with obsessive-compulsive disorder, the Bergen 4-day treatment group was found to be superior to internet-based cognitive behavioral therapy in reducing obsessive-compulsive symptoms, with large effects at 3-month follow-up.

**Meaning:**

These findings suggest the Bergen 4-day treatment is effective for patients with obsessive-compulsive disorder.

## Teaser

This randomized clinical trial evaluates the efficacy of the Bergen 4-day treatment compared with internet-based cognitive behavioral therapy in reducing obsessive-compulsive disorder symptom severity.

## Introduction

Obsessive-compulsive disorder (OCD) affects 1% to 3% of the population and is associated with substantial functional impairment, reduced quality of life, and societal burden worldwide.^1,2,3,4^ Exposure and response prevention (ERP) is the recommended first-line psychological treatment^5,6^ and has demonstrated robust efficacy across meta-analyses.^7,8^ However, access to and usage of ERP remain limited due to shortages of trained therapists, long waiting times,^9^ and treatment refusal and dropout,^10^ further limiting its scalability in routine care. These challenges have recently been highlighted in a report by the International OCD Foundation that points out persistent barriers to access to evidence-based OCD care.^11^ As mental health systems increasingly emphasize efficiency and accessibility, optimizing delivery formats while preserving clinical benefits is a priority.^12^

The Bergen 4-day treatment (B4DT) was developed as a concentrated ERP intervention (cERP) with an intention-focused approach that is delivered over 4 consecutive days following a brief preparatory phase.^13^ By compressing treatment traditionally delivered over several months into 4 days, B4DT aims to enhance efficiency and accessibility while maintaining therapeutic intensity.

A key innovation is the Leaning into Anxiety (LET) protocol,^13^ in which patients are guided to intentionally engage with feared stimuli. This approach emphasizes willingness and approach behavior, potentially altering how patients relate to anxiety during and beyond exposure.

Studies conducted primarily in Scandinavia report high response and remission rates.^14,15,16,17,18^ However, the existing evidence is dominated by uncontrolled designs. Only 1 randomized clinical trial (RCT) has evaluated B4DT, comparing it with waiting list and structured self-help conditions.^19^ Although that study provided important proof-of-concept evidence, it did not address whether B4DT confers incremental benefit over scalable digital cognitive behavioral therapy (CBT) interventions that are increasingly being implemented within stepped-care systems. Moreover, waiting list comparisons may inflate estimated treatment effects relative to more stringent controls,^20^ and psychotherapy trials more broadly can be susceptible to researcher allegiance effects associated with observed outcomes.^21,22^

Determining whether a concentrated, specialist-delivered intervention, such as B4DT, confers meaningful advantages over scalable alternatives is central to contemporary stepped-care models. Internet-based CBT (iCBT) is an evidence-based and cost-effective approach that is increasingly being used, even with minimal guidance.^23,24^ As digital-first pathways expand, comparative effectiveness data are needed to guide resource allocation, particularly given that digital interventions often yield smaller effects and have higher dropout rates than therapist-guided approaches.^25^

Methodological considerations further complicate the interpretation of findings in prior B4DT studies. In several studies, baseline was assessed immediately before the core exposure phase, following a preparatory period. Because B4DT includes structured preparation, defining baseline at that point may obscure treatment-related change and limit comparability with standard ERP trials, which define baseline before initiation of therapeutic procedures. In addition, this approach may introduce sampling bias as individuals who discontinue during the preparatory phase may not be represented.

This RCT addressed these gaps by comparing B4DT with an active, evidence-based iCBT intervention.^26^ Baseline assessment was conducted prior to the preparatory phase to capture the full intervention trajectory and align with established ERP methodology. This study provided the first evaluation of B4DT against an active, evidence-based treatment and generated clinically actionable evidence to inform treatment selection, implementation strategies, and health system resource allocation in OCD care.

The primary hypothesis was that participants receiving B4DT would demonstrate greater reductions in OCD symptom severity from baseline to 3-month follow-up compared with those receiving the active control condition (iCBT) as measured by the Yale-Brown Obsessive Compulsive Scale (Y-BOCS). Secondary hypotheses concerned the superiority of B4DT compared with iCBT in all secondary outcomes, including quality of life, depressive symptoms, global functioning, response and remission rates, and treatment acceptability.

## Methods

### Study Design

This single-center RCT was conducted at the University Medical Center Hamburg-Eppendorf, Germany. Between May 8, 2024, and August 8, 2025, participants were enrolled. The study compared B4DT (adapted for use in Germany) with an active control condition consisting of an iCBT program for OCD.^26^ The protocol was prospectively registered and approved by the local ethics committee (No. LPEK-0747). All participants provided written informed consent. The trial was conducted and reported in accordance with the Consolidated Standards of Reporting Trials (CONSORT) reporting guidelines. The study protocol was compiled based on preregistered materials and is available in Supplement 1. Patients were not involved in the design, conduct, or reporting of the study. Patient organizations (eg, German Society for OCD, OCD Land) assisted with the recruitment.

Assessments were conducted at baseline (t0), 6 weeks later (t1), posttreatment (t2; approximately 8 weeks after baseline), and 3-month follow-up (t3). A 12-month follow-up assessment (t4) was added during the course of the study to assess longer-term outcomes and will be analyzed separately from the primary end point analyses.

### Participants

Eligible participants were adults aged 18 to 75 years with a primary diagnosis of OCD (according to the *Diagnostic and Statistical Manual of Mental Disorders* [Fifth Edition]), verified using the Diagnostic Interview for Anxiety, Mood, and OCD and Related Neuropsychiatric Disorders (DIAMOND).^27^ The DIAMOND interview was administered to assess eligibility and identify any exclusion criteria. Diagnostic modules not relevant to the study objectives or the eligibility assessment (eg, neurodevelopmental disorders, somatic symptom and related disorders, and selected other disorders) were omitted. Participants were not required to be treatment naive, reflecting routine clinical practice. Previous ERP experience was recorded at baseline. If patients were taking medication, stable psychopharmacological treatment was required for at least 6 weeks prior to study entry. Exclusion criteria were acute suicidality, impaired intellectual functioning, current psychotic disorder (schizophrenia, bipolar disorder), autism spectrum disorder, substance dependence, insufficient German language proficiency, contraindication for in vivo exposure (eg, severe cardiovascular disease), and neurological disorder associated with secondary OCD.

### Randomization

The random allocation sequence was generated using computer-generated block randomization (block size = 8). No matching, stratification, or minimization procedures were applied. After baseline assessment (t0), participants were assigned in a 1:1 ratio to B4DT or iCBT according to the predefined allocation sequence via email. The person responsible for allocation was not involved in baseline assessments. Outcome assessors were blinded to treatment allocation.

### Interventions

#### cERP Therapy

The intervention consisted of 2 preparatory individual 50-minute sessions with each participant followed by 4 consecutive days of B4DT delivered in small groups (typically 4 patients) by trained therapists with a 1:1 patient-to-therapist ratio. During the pilot implementation,^28,29^ the therapists were trained and certified by the Bergen team. Subsequently, newly appointed therapists were trained and supervised by certified B4DT therapists within the Hamburg team. Throughout the trial, all treatment groups were supervised by 3 B4DT group leaders (L.J., J.S., and A.H.Y.) to ensure treatment fidelity.

Day 1 focused on manualized psychoeducation and individualized exposure planning in the group. On days 2 and 3, participants completed concentrated, individually tailored in vivo exposure exercises across multiple OCD-relevant situations, supported by therapists throughout each day, and self-guided ERP exercises in the evening and the following morning. Group meetings were held to review progress and challenges and to share learnings. On the afternoon of day 3, a psychoeducation session for relatives was conducted. Day 4 focused on consolidation of treatment gains, relapse prevention, and planning of self-guided ERP for the upcoming 30 days. An individual booster session was scheduled approximately 3 months after treatment.

The intervention was based on the original Bergen B4DT protocol and retained its core therapeutic principles, treatment structure, therapist-to-patient ratio, and exposure-based approach. Minor contextual adaptations, including refinements to preparatory sessions and culturally adapted patient materials, were made to support implementation in the German health care context.

#### iCBT

Participants in the control group received access to an unguided, OCD-specific iCBT program consisting of eight modules.^26,30^ The program was delivered in its fixed, prestructured version, which comprises psychoeducation and CBT-based exercises targeting obsessions and compulsions, including a dedicated ERP module, alongside modules on mindfulness, self-esteem, and social skills. The intervention was entirely self-guided, although participants could contact the study team for technical support if needed. Participants in both groups were allowed to continue treatment as usual, including pharmacological treatment and outpatient psychotherapy.

### Outcomes

The primary outcome was change in obsessive-compulsive symptom severity as measured by the Y-BOCS^31^ from t0 to t3. Secondary outcomes were Y-BOCS–defined response and remission rates and changes in self-reported obsessive-compulsive symptoms (Obsessive-Compulsive Inventory–Revised [OCI-R]^32^), depressive symptoms (Patient Health Questionnaire–9 [PHQ-9]^33^), quality of life (World Health Organization Quality of Life–BREF, global item [WHOQOL-BREF]^34^), self-esteem (Rosenberg Self-Esteem Scale [RSE]^35^), experiential avoidance (Brief Experiential Avoidance Questionnaire [BEAQ]^36^), obsessive beliefs (Obsessive Beliefs Questionnaire–44 [OBQ-44]^37^), and global functioning (Global Assessment of Functioning [GAF]^38^). Treatment satisfaction was assessed using the Client Satisfaction Questionnaire–8 (CSQ-8^39^). Additional prespecified measures, including adverse effects assessed using the Inventory for the Assessment of Negative Effects of Psychotherapy, were not part of the confirmatory testing and are therefore not included in the current efficacy analyses.

### Outcome Assessors and Interrater Reliability

The DIAMOND diagnostic interview and Y-BOCS assessments were conducted by advanced psychology students and graduate psychologists who were blinded to treatment allocation. Follow-up assessments at t3 were conducted in person whenever feasible. However, to maximize retention and accommodate participant preferences, secure video conferencing was used when an in-person assessment was not feasible. Telephone assessments were used only in exceptional cases when technical difficulties prevented video-based assessment. All raters completed a standardized training program consisting of an e-learning curriculum, practice interviews with patients, formal interrater reliability testing, and a competency examination before independently conducting study assessments. Throughout the trial, raters participated in regular supervision to ensure adherence to standardized assessment procedures and maintain rating quality. Interrater reliability for the Y-BOCS was good (intraclass correlation coefficient = 0.85; 95% CI, 0.63-0.96; *P* < .001).

### Sample Size

Sample size calculations assumed a conservative effect size of Cohen *d* = 0.52, requiring 120 participants to achieve 80% power with α = .05. Although the pilot study yielded an effect size of Cohen *d* = 0.62 (mean difference: 4.12^28^), a more conservative effect size was assumed for the RCT to account for the greater heterogeneity expected.

### Statistical Analysis

All analyses were conducted according to the intention-to-treat principle (ITT) and included all randomized participants. Primary and secondary continuous outcomes were analyzed using mixed models for repeated measures (MMRM), which account for the correlation of repeated measurements within participants and use all available data. For each outcome, models included fixed effects for treatment group (B4DT vs iCBT), time (t1, t2, and t3), the group × time interaction, and the baseline value (t0) of the respective outcome as a covariate. Baseline was included as a covariate rather than as a repeated time point to improve precision and control for baseline differences between groups. As analyses of secondary outcomes were intended to provide supportive and exploratory information on the broader clinical treatment effects, no adjustment for multiple testing was applied.

Within-subject correlations across repeated measurements were modeled using an unstructured covariance matrix. Estimated marginal means and corresponding standard errors were derived for each treatment group at each time point. Between-group differences at t2 and t3 were obtained from pairwise comparisons of estimated marginal means and reported as least squares mean differences (LSMD), with 95% CIs.

Standardized mean differences (Hedges *g*) were calculated by dividing the LSMD by the square root of the model-based residual variance at the relevant time point. Model-based within-group changes from t1 to t3 were derived from pairwise comparisons of estimated marginal means within each treatment group using the same MMRM as the between-group LSMDs.

Effect sizes (*g*) were interpreted using conventional thresholds: 0.2 (small), 0.5 (moderate), and 0.8 (large). We calculated 95% CIs for Hedges *g* using the CIs of the LSMD. Missing data were handled using MMRM under the assumption that data were missing at random.

As a post hoc sensitivity analysis, all mixed-effects models were repeated, with the inclusion of randomization block as an additional fixed effect to account for potential cohort-related effects. Complete-case analyses using analysis of covariance models adjusted for baseline values were also performed as supplementary sensitivity analyses to assess the robustness of the findings. Because these analyses were exploratory sensitivity analyses, effect sizes were reported as partial eta squared (η_p_^2^), the conventional effect size measure for analysis of variance and analysis of covariance. Analyses were performed using IBM SPSS Statistics, version 31 (IBM Corp.). Treatment response was defined as a greater than or equal to 35% reduction in Y-BOCS score and remission as a score less than or equal to 12.^40^ Statistical significance was set at *P* < .05, and all tests were 2-tailed.

## Results

### Participants

A total of 119 participants (mean [SD] age = 33.5 [10.1] years; 79 women [66.4%]) were randomized to B4DT (n = 60) or iCBT (n = 59). Baseline characteristics are shown in Table 1. Of the individuals who declined to participate, 48 (59.3%) were women, a proportion comparable to that of the enrolled sample (66.4%). All 119 randomized participants were included in the primary ITT analysis. Participant flow and numbers completing each assessment are presented in Figure 1.

**Table 1.** Demographics of the Participants at Baseline Assessment (t0)

Characteristics	Participants, No. (%)		
	B4DT (n = 60)	iCBT (n = 59)	Total sample (N = 119)
Gender			
Women	42 (70.0)	37 (62.7)	79 (66.4)
Men	18 (30)	22 (37.3)	40 (33.6)
Gender diverse^a^	0	0	0
Age, mean (SD), y	32.44 (9.76)	34.51 (10.45)	33.47 (10.12) [range, 18-72]
Education, mean (SD), years of school	12.70 (1.85)	12.44 (1.26)	12.57 (1.59)
OCD content, related to			
Contamination	35 (58.3)	30 (50.8)	65 (54.6)
Checking	51 (85)	46 (78)	97 (81.5)
Taboo	12 (20)	13 (22)	25 (21)
No. of inpatient treatments (lifetime), mean (SD)	0.65 (1.29)	1.03 (1.71)	0.84 (1.52)
Previous ERP experience^b^^,^^c^			
None	12 (20.3)	14 (23.7)	26 (22.0)
Self-planned only	12 (20.3)	7 (11.9)	19 (16.1)
Therapist-planned	13 (22.0)	8 (13.6)	21 (17.8)
Therapist-assisted	22 (37.3)	30 (50.8)	52 (44.1)
Medication^b^			
Antidepressant	19 (32.2)	26 (44.1)	45 (38.1)
Combination (antidepressant and neuroleptic)	0	1 (1.7)	1 (0.8)
No psychotropic medication	40 (67.8)	32 (54.2)	72 (61.0)
Age of OCD onset, mean (SD), y	18.85 (9.51)	21.17 (12.30)	20.00 (11.00)
Duration of OCD, mean (SD), y	10.37 (8.51)	11.46 (7.97)	10.91 (8.23)

Abbreviations: B4DT, Bergen 4-day treatment; ERP, exposure and response prevention; iCBT, internet-based cognitive behavioral therapy; OCD, obsessive-compulsive disorder.

^a^ No participants identified as gender diverse; therefore, this category was not included in the analyses.

^b^ N = 118; 1 participant in the B4DT group did not provide the requested information.

^c^ Participants were classified according to the highest level of previous ERP experience (none, self-planned only, therapist-planned, therapist-assisted).

**Figure 1.**
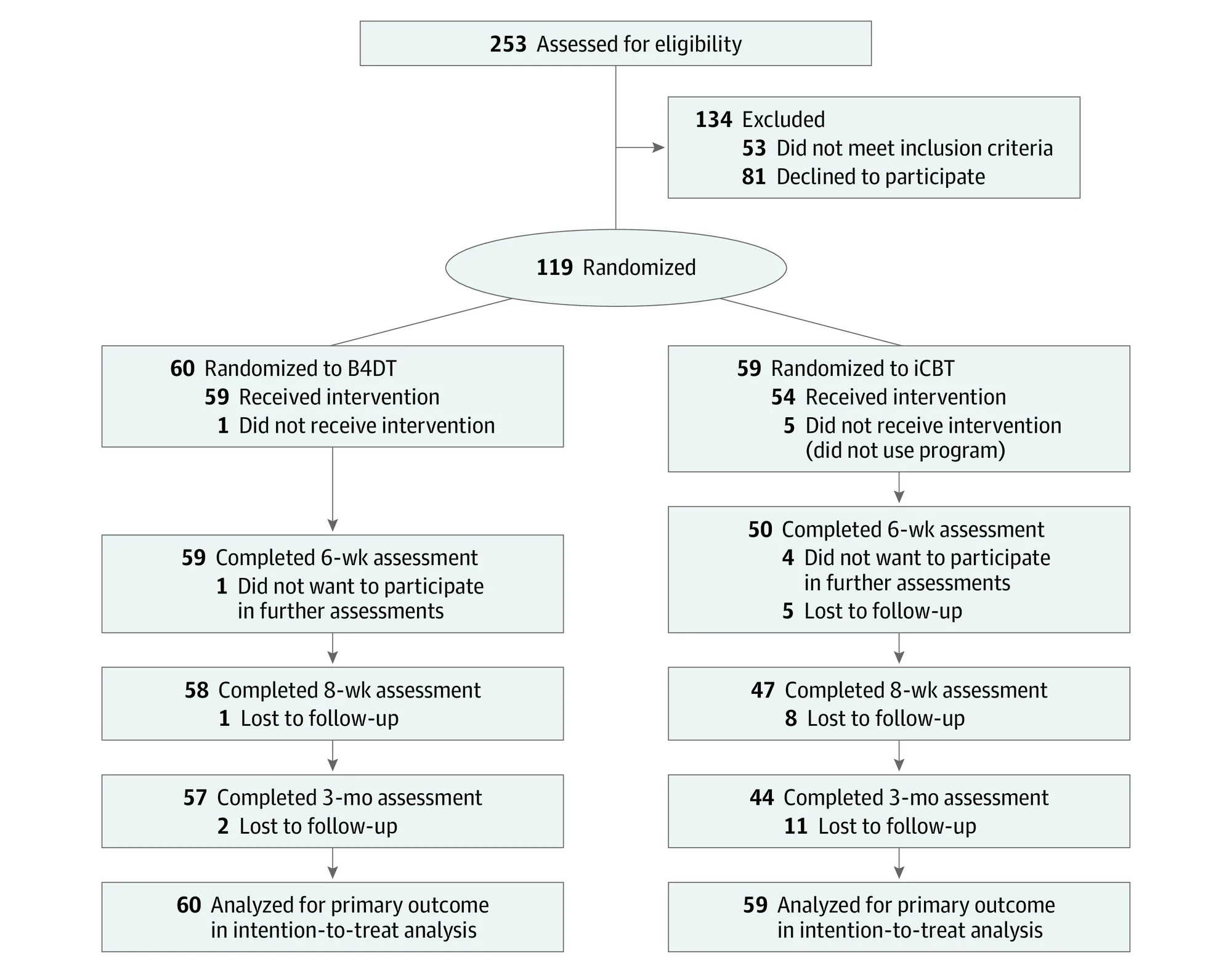
CONSORT Flow Diagram Flow of participants through the randomized clinical trial. B4DT indicates Bergen 4-Day Treatment;CONSORT indicates consolidated standards of reporting trials; iCBT, indicates internet-based cognitive behavioral therapy.

### Primary Outcome (Y-BOCS)

The primary ITT analysis, which included all 119 randomized participants, showed a significant group × time interaction for OCD severity measured with the Y-BOCS, *F*_(2, 103.07)_ = 50.45; *P* < .001. Participants receiving B4DT showed significantly greater reductions at t2 than those receiving iCBT (mean [SE] difference, −7.86 [0.9]; 95% CI , −9.70 to −6.02]; *g* = −1.64 [95% CI, −2.02 to −1.26]) and at t3 (mean [SE] difference, −5.61 [1.1]; 95% CI, −7.83 to −3.39]; *g* = −0.99 [95% CI, −1.38 to −0.60]) (Figure 2 and Table 2).

**Figure 2.**
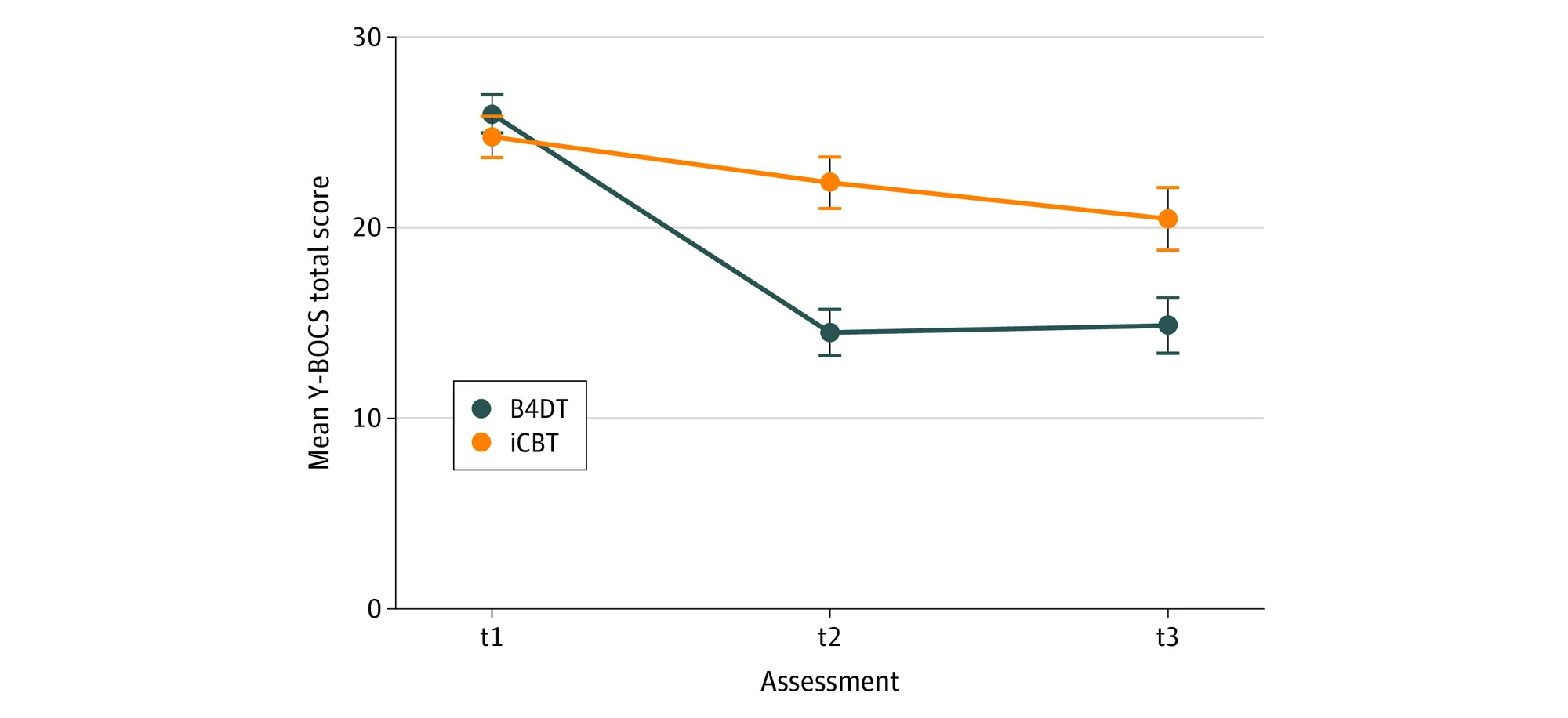
Line Chart of Change in Obsessive-Compulsive Symptom Severity Over Time Mean Yale-Brown Obsessive Compulsive Scale (Y-BOCS) total scores for participants receiving Bergen 4-Day Treatment (B4DT) or internet-based cognitive behavioral therapy (iCBT) at 6 weeks (t1), 8 weeks (t2), and 3-month follow-up (t3).

**Table 2.** Baseline Observed Means and Adjusted Follow-Up Outcomes With Standardized Effect Sizes and Results of Mixed Models for Repeated Measures^a^

Outcome and group	Baseline, mean (SD)	t1, mean (SE)	t2			t3			*P* value (group × time)^d^	Within-group difference, t1 − t3 (SE) [95% CI]
			Mean (SE)	LSMD (95% CI)^b^	Hedges g (95% CI)^c^	Mean (SE)	LSMD (95% CI)^b^	Hedges g (95% CI)^c^		
Y-BOCS (primary)										
B4DT	26.13 (4.63)	25.97 (0.51)	14.50 (0.62)	−7.86 (−9.70 to −6.02)	−1.64 (−2.02 to −1.26)	14.86 (0.74)	−5.61 (−7.83 to −3.39)	−0.99 (−1.38 to −0.60)	<.001	11.11 (0.80) [9.53 to 12.69]
iCBT	27.25 (6.42)	24.76 (0.55)	22.36 (0.69)	NA	NA	20.46 (0.84)	NA	NA		4.30 (0.89) [2.53 to 6.07]
Self-rated OCD severity (OCI-R)										
B4DT	28.43 (10.35)	25.09 (0.76)	10.26 (0.94)	−11.79 (−14.66 to −8.92)	−1.63 (−2.03 to −1.24)	12.76 (0.96)	−7.54 (−10.44 to −4.63)	−1.03 (−1.43 to −0.63)	<.001	12.32 (1.07) [10.20 to 14.45]
iCBT	24.37 (11.22)	23.55 (0.84)	22.05 (1.09)	NA	NA	20.30 (1.10)	NA	NA		3.25 (1.22) [0.84 to 5.66]
Depression (PHQ-9)										
B4DT	9.30 (5.52)	7.90 (0.47)	4.12 (0.49)	−2.93 (−4.42 to −1.45)	−0.78 (−1.18 to −0.39)	5.26 (0.59)	−2.33 (−4.11 to −0.55)	−0.52 (−0.92 to −0.12)	<.001	2.64 (0.67) [1.31 to 3.98]
iCBT	9.19 (4.55)	7.58 (0.52)	7.06 (0.57)	NA	NA	7.59 (0.68)	NA	NA		−0.01 (0.77) [−1.54 to 1.52]
Quality of life (WHOQOL-BREF)										
B4DT	3.00 (0.92)	3.02 (0.08)	3.89 (0.09)	0.58 (0.31 to 0.84)	0.86 (0.46 to 1.26)	3.62 (0.10)	0.48 (0.17 to 0.79)	0.61 (0.22 to 1.01)	<.001	−0.60 (0.11) [−0.82 to −0.39]
iCBT	2.85 (0.94)	3.16 (0.09)	3.32 (0.10)	NA	NA	3.14 (0.12)	NA	NA		0.02 (0.12) [−0.23 to 0.27]
Global functioning (GAF)										
B4DT	63.13 (8.96)	61.96 (0.74)	74.08 (0.95)	9.69 (6.88 to 12.49)	1.34 (0.95 to 1.73)	75.41 (1.14)	6.50 (3.08 to 9.92)	0.75 (0.35 to 1.14)	<.001	−13.45 (1.17) [−15.77 to −11.14]
iCBT	59.42 (10.32)	62.31 (0.81)	64.39 (1.05)	NA	NA	68.91 (1.29)	NA	NA		−6.60 (1.32) [−9.21 to −3.98]
Obsessive beliefs (OBQ-44)										
B4DT	192.29 (46.26)	181.65 (3.32)	116.83 (5.15)	−38.50 (−54.26 to −22.75)	−1.00 (−1.41 to −0.59)	117.16 (5.32)	−40.98 (−57.19 to −24.76)	−1.03 (−1.44 to −0.62)	<.001	64.49 (6.07) [52.45 to 76.53]
iCBT	170.25 (44.11)	166.83 (3.72)	155.33 (5.97)	NA	NA	158.13 (6.14)	NA	NA		8.70 (6.93) [−5.05 to 22.45]
Self-esteem (RSE)										
B4DT	29.28 (6.02)	29.20 (0.47)	33.12 (0.52)	2.51 (0.95 to 4.08)	0.63 (0.24 to 1.03)	33.17 (0.62)	2.39 (0.52 to 4.26)	0.51 (0.11 to 0.91)	.002	−3.97 (0.65) [−5.26 to −2.68]
iCBT	28.64 (6.19)	29.38 (0.53)	30.60 (0.60)	NA	NA	30.78 (0.71)	NA	NA		−1.40 (0.75) [−2.89 to 0.08]
Experiential avoidance (BEAQ)										
B4DT	51.43 (10.35)	49.56 (0.96)	37.84 (1.32)	−9.26 (−13.25 to −5.27)	−0.92 (−1.32 to −0.53)	37.39 (1.38)	−9.05 (−13.24 to −4.85)	−0.86 (−1.26 to −0.46)	<.001	12.17 (1.34) [9.50 to 14.84]
iCBT	50.27 (11.10)	49.60 (1.07)	47.10 (1.51)	NA	NA	46.44 (1.59)	NA	NA		3.16 (1.56) [0.07 to 6.26]

Abbreviations: B4DT, Bergen 4-Day Treatment; BEAQ, Brief Experiential Avoidance Questionnaire; GAF, Global Assessment of Functioning; iCBT, internet-based cognitive behavioral therapy; LSMD, least squares mean difference; NA, not applicable; OBQ-44, Obsessive Beliefs Questionnaire–44; OCI-R, Obsessive-Compulsive Inventory–Revised; PHQ-9, Patient Health Questionnaire–9; RSE, Rosenberg Self-Esteem Scale; t1, 6-week assessment; t2, assessment at approximately 8 weeks; t3, assessment at 3-month follow-up; WHOQOL-BREF, World Health Organization Quality of Life (single item); Y-BOCS, Yale-Brown Obsessive Compulsive Scale.

^a^ Data are presented as mean (SD) at baseline and as estimated marginal means (SE) at t1, t2, and t3 derived from linear mixed models adjusted for baseline values.

^b^ LSMD represents B4DT minus iCBT at t2 and t3.

^c^ Standardized mean differences (Hedges g) were calculated by dividing the adjusted mean differences by the square root of the model-estimated variance at the corresponding follow-up assessment, applying a small-sample correction based on the Satterthwaite degrees of freedom. Confidence intervals for Hedges g were obtained by applying the same transformation to the confidence intervals of the adjusted mean differences.

^d^ *P* values correspond to the group × time interaction from the mixed model.

### Secondary Continuous Outcomes

Significant group × time interactions were also observed for self-reported obsessive-compulsive symptoms (OCI-R), depressive symptoms (PHQ-9), quality of life (WHOQOL-BREF), global functioning (GAF), obsessive beliefs (OBQ-44), self-esteem (RSE), and experiential avoidance (BEAQ). Across these measures, improvements consistently favored B4DT at both t2 and t3. Standardized effect sizes ranged from moderate to large (−1.63 to 1.34 at t2 and −1.03 to 0.75 at t3) (Table 2).

### Sensitivity Analyses

In complete-case analyses, results were consistent with the ITT analyses (see eTables 1 and 2 in Supplement 2). Results of the post hoc sensitivity analyses including randomization block as an additional fixed effect were largely unchanged across the primary and secondary outcomes (see eTable 3 in Supplement 2).

### Treatment Response, Remission, and Acceptability

Mean response rates were higher with B4DT than with iCBT. From t0 to t2, mean response rates were 44.03% (n = 58) for B4DT vs 14.56% (n = 47) for iCBT, and at t3 they were 42.67% (n = 57) vs 20.41% (n = 44), respectively.

At t2, 38 of 58 participants (65.5%) receiving B4DT met the predefined response criteria (≥35% reduction) compared with 4 of 47 (8.5%) receiving iCBT; at t3, response rates were 35 of 57 (61.4%) vs 11 of 44 (25.0%), respectively. Remission rates (≤12 total Y-BOCS score) were also higher among those receiving B4DT, with 20 of 58 participants (34.5%) vs 4 of 47 (8.5%) achieving remission at t2 and 24 of 57 (42.1%) vs 6 of 44 (13.6%) at t3. Mean (SD) CSQ-8 scores were higher for those receiving B4DT (n = 56) than iCBT (n = 39) at t2 (30.77 [2.20] vs 19.44 [4.79]; *t*(93) = 15.52; *P* < .001; Hedges *g* = 3.21 [95% CI, 2.59-3.82]) and t3 (29.45 [3.58] vs 19.51 [5.23]; *t*(93) = 11.00; *P* < .001; Hedges *g* = 2.28 [95% CI, 1.75-2.79]).

## Discussion

In this RCT, B4DT was associated with greater reductions in OCD symptom severity compared with an evidence-based, unguided iCBT program, making it the first RCT to evaluate B4DT against an active, evidence-based digital self-help intervention rather than a waiting list or minimal intervention control. Treatment effects of B4DT were large at posttreatment (t2) and remained robust at 3-month follow-up (t3), with consistent superiority across secondary outcomes. Furthermore, response and remission rates were higher and treatment satisfaction was markedly greater in the B4DT group. Together, these findings suggest that a concentrated, therapist-guided, intention-focused exposure format may offer greater clinical benefit than an evidence-based, unguided digital self-help intervention, even though the interventions differed considerably in therapist involvement and treatment intensity.

Nonetheless, participants receiving unguided iCBT also demonstrated clinically meaningful within-group improvements in OCD symptom severity and related outcomes, supporting such approaches as a valuable component of modern treatment pathways. These improvements continued through the 3-month follow-up, suggesting that participants may have continued to engage with and benefit from the self-guided intervention after the formal treatment period. Importantly, these findings indicate that the observed superiority of B4DT cannot simply be attributed to comparison with an inactive control condition such as a waiting list but rather reflects greater benefit relative to an active, evidence-based digital self-help intervention that itself was associated with clinically meaningful within-group improvement. At the same time, the magnitude of these effects was generally smaller than those reported for therapist-guided iCBT.^23^ Given the substantial treatment gap in OCD due to barriers associated with access, cost, and workforce limitations, scalable digital interventions play an important role in expanding access to care. Especially within contemporary stepped-care models, digital mental health interventions are increasingly being incorporated into routine care worldwide, although their level of integration varies considerably across health care systems. Their broader implementation holds considerable potential to address unmet treatment needs through accessibility, flexibility, and cost-effectiveness. Thus, rather than representing competing approaches, digital interventions, conventional therapist-delivered ERP, and concentrated formats such as B4DT may be best understood as complementary components within stepped-care models. iCBT may serve as a low-threshold, first-line intervention, whereas therapist-delivered ERP and, for certain patients, concentrated formats such as B4DT may provide more intensive treatment options depending on the patients’ clinical needs, preferences, and available health care resources.

The present findings that a concentrated, therapist-delivered intervention was associated with substantially greater clinical improvement than an unguided digital self-help intervention suggest that B4DT may be particularly beneficial for patients requiring rapid and pronounced symptom reduction. Importantly, the core therapeutic changes were achieved within a 4-day treatment period. From a health systems perspective, such approaches may increase treatment capacity and patient throughput while maintaining clinical effectiveness. However, the present trial was designed to compare B4DT with an evidence-based unguided digital intervention rather than therapist-delivered CBT. Consequently, direct comparisons with standard face-to-face ERP require future head-to-head trials.

Although the B4DT has demonstrated promising clinical outcomes, its implementation does present challenges. The model requires concentrated therapist resources over 4 consecutive days and a 1:1 patient-to-therapist ratio, which may hinder its implementation in some health care systems and service settings. However, comparisons between ERP delivery formats should consider the overall therapist time required per completed treatment episode and the total treatment dose rather than focusing solely on therapist involvement during the acute treatment phase.

In Germany, standard outpatient CBT for OCD is typically delivered over several months and may comprise up to 24 sessions, with the possibility of extension to 60 sessions, while international data indicate that intensive OCD treatment programs last 8 to 9 weeks on average.^41^ Thus, B4DT should be viewed as 1 of several evidence-based ERP delivery formats, each with distinct strengths, limitations, and indications depending on the local service organization.

A key question arising from these findings concerns the mechanisms underlying the large treatment effects observed with B4DT. Several factors likely interact. First, the concentrated delivery of exposure over consecutive days may reduce opportunities for avoidance and promote sustained engagement with feared stimuli for both patients and therapists. From an inhibitory learning perspective,^42^ this temporal compression may increase the frequency and salience of expectancy violations, thereby strengthening the formation of new, nonthreatening associations. Second, unlike the unguided iCBT comparator, B4DT offers continuous therapist support and a 1-to-1 patient-to-therapist ratio, which may reduce avoidance, facilitate adherence, and optimize the quality of ERP.^43^ Third, the group format^10^ and inclusion of relatives^44^ may enhance motivation, social reinforcement, and generalization of treatment gains. Fourth, the intention-focused approach during exposure may shift patients’ relationship to anxiety from avoidance to an active stance, potentially increasing engagement and the transfer of learning beyond the treatment context.^13^ This integrated perspective suggests that the effectiveness of B4DT may be attributable not to a single component but rather to the synergistic interactions among temporal, contextual, and therapeutic variables. Finally, beyond symptom reduction, the high levels of treatment satisfaction observed in the B4DT group suggest that patients may experience the treatment as particularly engaging and meaningful. The combination of rapid symptom change, intensive therapist support, and an active, approach-oriented therapeutic stance may enhance patients’ sense of agency and mastery. These experiential factors may play a critical role in adherence, acceptability, and follow-up outcomes.

In contrast to earlier studies reporting high response and remission rates—often based on uncontrolled or nonblinded designs that may have inflated effect estimates—the present RCT provided a more rigorous estimate of the added clinical benefit of B4DT compared with an active digital self-help intervention. In addition, baseline assessment was conducted prior to the preparatory phase, allowing for a clearer delineation of treatment-related change and reducing potential bias introduced by early intervention effects. This more precise temporal framing enhanced the interpretability of outcomes and improved comparability with standard ERP trials. Compared with prior studies of B4DT, particularly the uncontrolled trials conducted in Scandinavian settings reporting response rates of approximately 80% and remission rates of around 70%,^16,17^ the present independent RCT found lower, although still substantial, response (61%) and remission (42%) rates at 3-month follow-up. Such differences are expected as uncontrolled implementation studies commonly yield larger treatment effects than more rigorous randomized evaluations. This discrepancy may also reflect differences in sample characteristics, including age of onset, demographic composition, and illness history, suggesting that the current study included a more heterogeneous clinical population than the earlier trials, which often applied more selective inclusion criteria. Notably, the only other RCT^19^ on B4DT excluded patients with prior CBT, whereas participants in the present trial were not required to be treatment naïve. Most participants had previous ERP experience, resulting in a more clinically representative sample than in earlier B4DT studies. Still, symptom improvements in the B4DT group were comparable to those observed in a recent German pilot trial, in which 54% were in remission at 3-month follow-up^28,29^ and 51% at 12-month follow-up^45^ and were more closely aligned with the 47% to 56% remission rates reported in a Scandinavian sample of difficult-to-treat OCD patients.^46^ Importantly, although outcome assessments in prior studies were often conducted by raters not directly involved in the treatment, full rater blinding cannot be ensured in uncontrolled designs, and expectancy effects (eg, Rosenthal effects) may have contributed to inflated estimates. By contrast, the methodological rigor of the current trial suggests that the present findings provide a more conservative and generalizable estimate of B4DT effectiveness in routine clinical populations. The rate of refusal to participate further highlights the need for future research examining determinants of participation in intensive exposure-based treatments and strategies to improve treatment engagement.

### Limitations

Several limitations should be considered. First, the study was conducted at a single center, which may limit generalizability. Second, follow-up was limited to 3 months; longer-term outcomes (in progress) are needed to determine the durability of treatment effects. Third, participants and therapists were not blinded to treatment allocation, which is inherent to psychotherapy trials but may introduce expectancy effects. Fourth, secondary outcome analyses were exploratory and not controlled for multiple testing. Fifth, because age and clinical characteristics were not systematically collected from individuals who declined to participate, assessment of potential selection bias was limited to gender distribution, which was comparable between participants and individuals who declined to participate. Additionally, adherence to the iCBT program may have varied across participants, which could have been associated with treatment outcomes and should be considered when interpreting outcomes. An additional limitation is that participants were allowed to receive concomitant treatment as usual, including outpatient psychotherapy; thus their potential contribution to outcomes cannot be fully disentangled. Future research should examine the long-term and cost-effectiveness of B4DT, as well as its implementation in routine care settings. Direct comparisons of concentrated B4DT with an equivalent dose of therapist-delivered ERP delivered over a longer period are needed to determine whether treatment intensity, rather than treatment content alone, contributes to clinical outcomes. In addition, hybrid or sequential treatment models combining digital and intensive approaches may further enhance outcomes while maximizing efficiency.

## Conclusions

In this RCT of 119 adults with OCD, B4DT was associated with greater and more sustained reductions in OCD symptom severity compared with an established iCBT intervention. Rather than representing competing approaches, these findings suggest the complementary roles of the 2 interventions within stepped-care frameworks. iCBT may serve as an accessible first-line treatment, whereas B4DT may offer a more intensive therapist-delivered second-line option for individuals requiring more intensive care. Integrating the 2 approaches has the potential to improve access, optimize resource allocation, and enhance overall treatment outcomes in OCD.

## Notes

Supplement 1. Trial Protocol

Supplement 2. eTable 1. Means (Standard Deviations) of Primary and Secondary Outcome Parameters for the Complete-Case Sample eTable 2. Complete-Case Sensitivity Analyses Using Repeated-Measures ANOVA and ANCOVA eTable 3. Sensitivity Analysis Including Randomization Block as an Additional Fixed Effect: Baseline Means, Adjusted Outcomes, Standardized Effect Sizes, and Mixed Models for Repeated Measures (MMRM)

Supplement 3. Data Sharing Statement
