# Supplementary material for: Concentrated Exposure Therapy vs Internet-Based Cognitive Behavioral Therapy for Obsessive-Compulsive Disorder: A Randomized Clinical Trial
Source: JAMA Netw Open. 2026 Oct 19;9(10):e2639281. doi: 10.1001/jamanetworkopen.2026.39281 (PMC13681233; doi:10.1001/jamanetworkopen.2026.39281)
Supplement: Supplementary file 1 [file jamanetwopen-e2639281-s001.pdf]

This protocol was compiled post hoc based on the preregistration, ethics application, and grant proposal and reflects the prespecified study procedures and analysis plan.

Evaluation of the Hamburg version of concentrated exposure therapy for people with Obsessive-Compulsive Disorder: A randomized controlled trial

Acronym/abbreviation of the study

HERO (Hamburg concentrated Exposure Response Prevention treatment for OCD)

Background and Rationale

Short, concentrated exposure treatments (cET), such as the "Bergen 4-day treatment" (B4DT),[1,2,3] represent an innovative method for improving cost and time efficiency in the treatment of obsessive-compulsive disorder (OCD). Patients receive individualized exposure therapy over the course of a few consecutive days. Studies from Norway show high remission rates of approximately 70% for B4DT, which remain stable over four years.[2,3] A pilot randomized controlled trial from Norway also showed that 94% of patients in the B4DT group responded to treatment, compared to 12% in the control group.[4] The extent to which these effects can be replicated in clinical settings in other countries remains unclear.[5]

In Germany, the B4DT was first tested by the applicants in a pilot study (ethical approval #LPEK-0512). Among the 33 patients, a large effect size was observed in the improvement on the Yale-Brown Compulsive Obsessive Scale (Y-BOCS) over the treatment period, with Cohen's d = 2.04, 95% CI [1.431, 2.637]. Three months after treatment, 15 individuals (47%) were in remission. Based on the experiences from the pilot study, necessary adaptations for application in the German setting were derived.

Objectives

Evaluation of the efficacy of the Hamburg version of the "Bergen 4-day Treatment" (B4DT) compared with internet-based cognitive behavioral therapy (iCBT) in reducing OCD symptom severity.

Hypotheses

The primary hypothesis was that participants receiving B4DT would demonstrate greater reductions in OCD symptom severity from baseline to 3-month follow-up compared with those receiving the active control condition (iCBT) as measured by the Yale-Brown Obsessive-Compulsive Scale (Y-BOCS). [8]

Secondary hypotheses concerned superiority of B4DT compared to iCBT in self-reported OCD symptom severity (OCI-R),[9] dysfunctional cognitions (OBQ-44),[10] quality of life,[13] depressive symptoms (PHQ-9),[11] experiential avoidance (BEAQ),[14] global functioning (GAF),[15] self-esteem (RSE),[12] response and remission rates, [28] and treatment acceptability (CSQ-8).[24]

Study Design (RCT)

Monocentric, interventional, randomized clinical two-arm trial (1:1 allocation)

Participants

Sample size calculation

The calculated sample size of 120 patients (60 per group) is based on an expected average effect size of $d = 0.52$ (at a 2-sided $\alpha = 0.05$ and $\beta = 0.80$)

Inclusion criteria

- Gender: All
- Minimum Age: 18 Years
- Maximum Age: 75 Years
- Diagnosed with obsessive-compulsive disorder (ICD-10: F 42)
- Willingness and consent to participate in the study (informed consent)
- Stable pharmacological treatment before treatment start (≥ 6 weeks)

Exclusion Criteria

- Acute suicidality
- Impaired intelligence
- Psychotic symptoms (incl. schizophrenia and bipolar disorder)

- Autism
- Substance dependence
- Insufficient German language skills
- Contraindication for in vivo exposure (e.g. cardiological contraindications)
- Neurological disorders that can trigger secondary compulsions

Interventions

Patients were randomized to Concentrated Exposure Treatment (cET, that is, B4DT, including preparatory sessions) or iCBT (8 modules), with comparable overall treatment time (8 weeks).

Arm 1:

Concentrated exposure treatment (cET): cET includes two pre-treatment sessions (preparation) and 4 consecutive treatment days (treatment week), which are conducted in a group of approx. 4 patients by the same number of therapists. The first day in the group setting is dedicated to manualized psychoeducation and planning the exposures. On the second and third day, the patients carry out individually tailored exposure training, supported by the therapists, in as many situations relevant to the OCD as possible. On the second and third day, the group meets to discuss the progress and challenges of the exposure training. On the afternoon of the third day, relatives and caregivers are invited to a psychoeducation session on understanding OCD and how they can best support the patient in their efforts to resist the compulsions. On the fourth day, patients are taught strategies for maintaining the changes, and self-guided exposures are planned for the following weeks.

Arm 2:

Unguided internet-based Cognitive Behavioral therapy (iCBT, “Zwanglos“): Participants in this arm receive access to the iCBT intervention, an online self-help program for coping with OCD symptoms, immediately after randomization. It includes information on OCD and interactive exercises on obsessive thoughts and compulsions, as well as mindfulness, self-esteem and social skills. The topics are divided into 8 modules of 45 minutes each. The program is specifically tailored to OCD and has already been found to be effective in previous studies.[6] Patients in this arm continue to have access to their standard treatment.

Assessments Points and Procedures

After participants have been screened and formally included in the study, symptoms and comorbidities (DIAMOND)[7] are assessed at baseline (t0) both through interviews (Y-BOCS [primary outcome]) and self-report measures (including, e.g., depression [BDI-II], quality of life [WHOQOL]). Additionally, secondary outcomes such as willingness to change [FEVER] and health economic data [FIMPsy] are collected. Participants are then randomly assigned to either the intervention group (cET) or the active control group (iCBT) with a 1:1 ratio, n = 60 per group. Participants in the intervention group will first receive two preparatory sessions, followed 6 weeks later by a 4-day, exposure-based therapy. Participants in the active control group will additionally receive access to a previously positively evaluated online self-help program for obsessive-compulsive symptoms [6].

For all patients, primary and secondary outcomes are assessed at baseline (t0) as well as 6 weeks (t1), 8 weeks (t2), and 3 months later (t3), and the subjective evaluation of treatment (ZUF-8) is recorded. An additional 12-month follow-up assessment (t4) was added during the course of the study to assess longer-term outcomes and will be analyzed and reported separately from the primary endpoint analyses.

In the intervention group, therapy-related parameters are assessed during treatment, e.g., group cohesion and therapeutic relationship (WAI). For patients living far away, diagnostic interviews are also available via video. As compensation, participants receive 30€ after the assessment at t3 and participants receive 30€ after the assessment at t4.

Outcome Measures

Primary outcome

- Yale-Brown Obsessive-Compulsive Scale (Y-BOCS):[8] The Y-BOCS is a clinician-based rating for the assessment of obsessive-compulsive symptoms. The primary endpoint is the change from baseline (t0) to follow-up (t3).

Secondary outcome

- Yale-Brown Obsessive-Compulsive Scale (Y-BOCS):[8] The Y-BOCS is an clinician-rated scale for the assessment of obsessive-compulsive symptoms. Response to treatment and remission rates are also measured with the Y-BOCS. In addition to the time points for the primary endpoint, the Y-BOCS is also carried out at t1, t2, and t4.

- Obsessive Compulsive Inventory-Revised (OCI-R):[9] The OCI-R records changes in self-reported obsessive-compulsive symptoms over different time points (t0, t1, t2, t3, t4).
- Obsessive-Beliefs-Questionnaire (OBQ-44):[10] The OBQ-44 measures changes in obsessive-compulsive dysfunctional attitudes (t0, t1, t2, t3, t4).
- Patients Health Questionnaire-Depression (PHQ-9):[11] The PHQ-9 is used to measure depression (t0, t1, t2, t3, t4).
- Rosenberg Self-Esteem Scale (RSES):[12] The RSES measures changes in self-esteem (t0, t1, t2, t3, t4).
- WHO Quality of Life-BREF (WHOQoL-Bref):[13] The WHOQoL-Bref examines changes in quality of life. We only use the global item (t0, t1, t2, t3, t4).
- Brief Experiential Avoidance Questionnaire (BEAQ):[14] The BEAQ is a self-report questionnaire and measures changes in experiential avoidance and psychological flexibility (t0, t1, t2, t3, t4).
- Global Assessment of Functioning (GAF):[15] The GAF assesses the general level of functioning. (t0, t1, t2, t3, t4)
- Intolerance of Uncertainty (IUS-12):[16] The IUS-12 is a self-report questionnaire and measures changes in intolerance of uncertainty over different time points (t0, t1, t2, t3).
- Readiness to change questionnaire (FEVER):[17] The FEVER is a self-assessment questionnaire and measures readiness to change (t0, t1, t2, t3).
- Valued Living Questionnaire (VLQ):[18] The VLQ examines values and constraints through compulsion in value-based action across different time points (t0, t1, t2, t3, t4).
- General Self-Efficacy Short Scale (ASKU):[19] The ASKU is a self-evaluation questionnaire and measures self-efficacy (t0, t1, t2, t3, t4).
- Clinical Perfectionism Questionnaire (CPQ-D):[20] The CPQ-D is a self-assessment questionnaire and measures perfectionism (t0, t1, t2, t3).
- Readiness for Exposure Therapy Questionnaire (FBE): The FBE measures readiness for exposure therapy at various points in time (t0, t1, t2, t3).
- Therapeutic Agency Inventory (TAI):[21] The TAI is a self-report questionnaire and measures readiness to change in therapy (t0, t1, t2, t3).

- Questionnaire on Responsibility in Therapy (FVT): The FVT measures responsibility in therapy at various points in time (t0, t1, t2, t3).
- Questionnaire on treatment expectations and attitudes (FTE): The FTE measures treatment expectations and attitudes at different points in time (t0, t1, t2, t3).
- Patient Questionnaire on Therapy Expectation and Evaluation (PATHEV):[22] The PATHEV measures hope for improvement and fear of change with regard to therapy (t0, t1).
- Questionnaire on the utilization of medical and non-medical care services for mental illness (FIMPsy):[23] The FIMPsy collects health economic data (t0, t2, t3, t4).
- Patient Satisfaction Questionnaire (ZUF-8):[24] The ZUF-8 assesses satisfaction with treatment (t2, t3).
- Inventory for the Assessment of Negative Effects of Psychotherapy (INEP):[25] The INEP records side effects of the therapy (t2, t3).

Only Intervention Group (B4DT)

- Pre and Post Session Questionnaire (PPSQ): This questionnaire records various aspects (such as self-efficacy expectation, expectation of treatment success, rating of tension, violation of expectations, group cohesion) on the compact days (at the beginning and end) (insession).
- Subscales "Bond" and “Task” of the Working Alliance Inventory - short revised (WAI-SR):[26] The WAI-SR measures the therapeutic relationship (before and after each of the exposure sessions on therapy days 2 and 3, each morning and afternoon (four sessions in total)). Both patients and therapist fill in the questionnaires. Additionally, the therapist estimates per session the ratio in which the patient practiced for themselves and records whether there was a change in the allocation between patient and therapist.
- Questionnaire on the satisfaction of relatives (ZUFA-8):[24] The ZUFA-8 is adapted and related to the relatives' group, which takes place once during the exposure week.
- Group Questionnaire (GQ-D):[27] The GQ-D examines group dynamics (t2).

Statistical Analysis Plan (SAP)

All analyses will be conducted according to the intention-to-treat (ITT) principle, including all randomized participants. Primary and secondary outcomes will be analyzed using linear mixed-effects models for repeated measures. The models will include fixed effects for treatment group (B4DT vs. iCBT), time (t1, t2, and t3), and the group × time interaction. The baseline value (t0) of the respective outcome will be included as a covariate.

The primary analysis focuses on outcomes at 3-month follow-up (t3). Analyses of the 12-month follow-up (t4) are pre-specified and hypothesis-driven but will be analyzed and reported separately from the primary endpoint analyses. No adjustment for multiple testing will be applied to the 12-month analyses, as these are reported separately. No interim analyses or stopping guidelines were prespecified.

Treatment response is defined as a reduction of at least 35% in the total Y-BOCS score, and remission is defined as a Y-BOCS score of 12 or below.[28]

Ethics and Approvals

The study protocol was approved by the Local Psychological Ethics Committee at the Center for Psychosocial Medicine (LPEK), University Medical Center Hamburg-Eppendorf, Germany.

Ethics Approval Number: #LPEK-0747, amendment (t4 assessment): #LPEK-0975

Date of Approval: 07.05.2024, amendment: (t4 assessment) 22.09.2025

The study was conducted in accordance with the Declaration of Helsinki. All participants provided written informed consent.

Trial Registration (pregistration)

German Clinical Trials Register (DRKS): DRKS00034236

Date of pregistration: 08.05.2024, amendment (t4 assessment): 30.09.2025

Data Handling / Data Protection

All study data will be collected solely for the purposes of this study. Personal identifiers (e.g., name, address, email) will be stored separately from study data and linked via a unique participant code. Pseudonymized electronic data will be stored on password-protected,

encrypted servers, and paper records will be kept in locked cabinets, with access restricted to authorized study personnel. Analyses will be conducted on pseudonymized data to prevent identification of individual participants. Data will be retained for 10 years in accordance with legal requirements and securely deleted thereafter. Any data sharing with third parties will occur only in anonymized form for scientific publications or audits. All procedures will comply with DSGVO regulations and ethical committee requirements, and participants will provide informed consent for the collection, storage, and use of their data

Protocol Amendments

An additional follow-up assessment at 12 months post-intervention (t4) has been added to evaluate the durability of treatment effects. This assessment will include all primary and secondary outcome measures. Analyses of the 12-month follow-up are pre-specified and hypothesis-driven but will be reported separately from the primary endpoint analyses. The primary endpoint remains unchanged. Ethics approval for this amendment was obtained prior to implementation (#LPEK-0975, 22.09.2025), and the trial registry (#DRKS00034236) was updated accordingly (30.09.2025).

References

1. Havnen A, Hansen B, Öst LG, Kvale G. Concentrated ERP delivered in a group setting: An effectiveness study. *J Obsessive-Compuls Relat Disord*. 2014;3(4):319-324. doi:10.1016/j.jocrd.2014.08.002

2. Hansen B, Kvale G, Hagen K, Havnen A, Öst LG. The Bergen 4-day treatment for OCD: four years follow-up of concentrated ERP in a clinical mental health setting. *Cogn Behav Ther*. 2019;48(2):89-105. doi:10.1080/16506073.2018.1478447

3. Hansen B, Hagen K, Öst LG, Solem S, Kvale G. The Bergen 4-Day OCD Treatment Delivered in a Group Setting: 12-Month Follow-Up. *Front Psychol*. 2018;9:639. doi:10.3389/fpsyg.2018.00639

4. Launes G, Hagen K, Öst LG, Solem S, Hansen B, Kvale G. The Bergen 4-Day Treatment (B4DT) for Obsessive-Compulsive Disorder: Outcomes for Patients Treated After Initial Waiting List or Self-Help Intervention. *Front Psychol*. 2020;11:982. doi:10.3389/fpsyg.2020.00982

5. Silver E, Isometsä E, Baryshnikov I, Heino H, Stenberg JH, Saarni SE. BERGEN 4-DAY TREATMENT (B4DT) FOR OBSESSIVE-COMPULSIVE DISORDER – AN OBSERVATIONAL PILOT STUDY OF A TREATMENT PROTOCOL IN FINLAND.

6. Schröder J, Werkle N, Cludius B, Jelinek L, Moritz S, Westermann S. Unguided Internet-based cognitive-behavioral therapy for obsessive-compulsive disorder: A randomized controlled trial. *Depress Anxiety*. 2020;37(12):1208-1220. doi:10.1002/da.23105

7. Tolin DF, Gilliam C, Wootton BM, et al. Psychometric Properties of a Structured Diagnostic Interview for *DSM-5* Anxiety, Mood, and Obsessive-Compulsive and Related Disorders. *Assessment*. 2018;25(1):3-13. doi:10.1177/1073191116638410

8. Goodman WK. The Yale-Brown Obsessive Compulsive Scale: I. Development, Use, and Reliability. *Arch Gen Psychiatry*. 1989;46(11):1006. doi:10.1001/archpsyc.1989.01810110048007

9. Foa EB, Huppert JD, Leiberg S, et al. The Obsessive-Compulsive Inventory: Development and validation of a short version. *Psychol Assess*. 2002;14(4):485-496. doi:10.1037/1040-3590.14.4.485

10. Obsessive Compulsive Cognitions Working Group. Development and initial validation of the obsessive beliefs questionnaire and the interpretation of intrusions inventory. *Behav Res Ther*. 2001;39(8):987-1006. doi:10.1016/S0005-7967(00)00085-1

11. Kroenke K, Spitzer RL, Williams JBW. The PHQ-9: Validity of a brief depression severity measure. *J Gen Intern Med*. 2001;16(9):606-613. doi:10.1046/j.1525-1497.2001.016009606.x

12. Rosenberg M. Rosenberg Self-Esteem Scale. Published online 1965. doi:10.1037/t01038-000

13. Skevington SM, Lotfy M, O'Connell KA. The World Health Organization's WHOQOL-BREF quality of life assessment: Psychometric properties and results of the international field trial. A Report from the WHOQOL Group. *Qual Life Res*. 2004;13(2):299-310. doi:10.1023/B:QURE.0000018486.91360.00

14. Gámez W, Chmielewski M, Kotov R, Ruggero C, Suzuki N, Watson D. The Brief Experiential Avoidance Questionnaire: Development and initial validation. *Psychol Assess*. 2014;26(1):35-45. doi:10.1037/a0034473

15. American Psychiatric Association. *Diagnostic and Statistical Manual of Mental Disorders*. 4th edition. 1994.

16. Carleton RN, Norton MAPJ, Asmundson GJG. Fearing the unknown: A short version of the Intolerance of Uncertainty Scale. *J Anxiety Disord*. 2007;21(1):105-117. doi:10.1016/j.janxdis.2006.03.014

17. Hasler G, Klaghofer R, Buddeberg C. Der Fragebogen zur Erfassung der Veränderungsbereitschaft (FEVER): Testung der deutschen Version der University of Rhode Island Change Assessment Scale (URICA). *PPmP - Psychother · Psychosom · Med Psychol*. 2003;53(9/10):406-411. doi:10.1055/s-2003-42172

18. Wilson KG, Sandoz EK, Kitchens J, Roberts M. The Valued Living Questionnaire: Defining and Measuring Valued Action within a Behavioral Framework. *Psychol Rec*. 2010;60(2):249-272. doi:10.1007/BF03395706

19. Beierlein C, Kovaleva A, Kemper CJ, Rammstedt B. Ein Messinstrument zur Erfassung subjektiver Kompetenzerwartungen: Allgemeine Selbstwirksamkeit Kurzskala (ASKU). 2012:24.

20. Roth I, Cludius B, Egan SJ, Limburg K. Evaluation of the factor structure and psychometric properties of the German version of the Clinical Perfectionism Questionnaire: The CPQ-D. *Clin Psychol Eur*. 2021;3(2):e3623. doi:10.32872/cpe.3623

21. Huber J, Nikendei C, Ehrenthal JC, Schauenburg H, Mander J, Dinger U. Therapeutic Agency Inventory. Published online September 13, 2021. doi:10.1037/t81916-000

22. Schulte D. Messung der Therapieerwartung und Therapieevaluation von Patienten (PATHEV). *Z Für Klin Psychol Psychother*. 2005;34(3):176-187. doi:10.1026/1616-3443.34.3.176

23. Grupp H, König HH, Riedel-Heller S, Konnopka A. FIMPsy – Fragebogen zur Inanspruchnahme medizinischer und nicht medizinischer Versorgungsleistungen bei psychischen Erkrankungen: Entwicklung und Verwendung. *Psychiatr Prax*. 2018;45(02):87-94. doi:10.1055/s-0042-118033

24. Attkisson CC, Zwick R. Client Satisfaction Questionnaire-8. Published online 1982. doi:10.1037/t50119-000

25. Ladwig I, Rief W, Nestoriuc Y. Welche Risiken und Nebenwirkungen hat Psychotherapie? - Entwicklung des Inventars zur Erfassung Negativer Effekte von Psychotherapie (INEP). *Verhaltenstherapie*. 2014;24(4):252-263. doi:10.1159/000367928

26. Hatcher RL, Gillaspy JA. Development and validation of a revised short version of the working alliance inventory. *Psychother Res*. 2006;16(1):12-25. doi:10.1080/10503300500352500

27. Bormann B, Burlingame GM, Strauß B. Der Gruppenfragebogen (GQ-D): Instrument zur Messung von therapeutischen Beziehungen in der Gruppenpsychotherapie. *Psychotherapeut*. 2011;56(4):297-309. doi:10.1007/s00278-011-0841-4

28. Mataix-Cols D, De La Cruz LF, Nordsletten AE, Lenhard F, Isomura K, Simpson HB. Towards an international expert consensus for defining treatment response, remission, recovery and relapse in obsessive-compulsive disorder. *World Psychiatry*. 2016;15(1):80-81. doi:10.1002/wps.20299
