# Supplementary material for: Concentrated Exposure Therapy vs Internet-Based Cognitive Behavioral Therapy for Obsessive-Compulsive Disorder: A Randomized Clinical Trial
Source: JAMA Netw Open. 2026 Oct 19;9(10):e2639281. doi: 10.1001/jamanetworkopen.2026.39281 (PMC13681233; doi:10.1001/jamanetworkopen.2026.39281)
Supplement: Supplementary file 2 [file jamanetwopen-e2639281-s002.pdf]

Supplemental Online Content

Jelinek L, Wille L, Scheunemann J, et al. Concentrated exposure therapy vs internet-based cognitive behavioral therapy for obsessive compulsive disorder. *JAMA Netw Open.* 2026;9(10):e2639281. doi:10.1001/jamanetworkopen.2026.39281

eTable 1. Means (Standard Deviations) of Primary and Secondary Outcome Parameters for the Complete-Case Sample

eTable 2. Complete-Case Sensitivity Analyses Using Repeated-Measures ANOVA and ANCOVA

eTable 3. Sensitivity Analysis Including Randomization Block as an Additional Fixed Effect: Baseline Means, Adjusted Outcomes, Standardized Effect Sizes, and Mixed Models for Repeated Measures (MMRM)

© 2026 Jelinek L et al. *JAMA Network Open*.

eTable 1. Means (Standard Deviations) of Primary and Secondary Outcome Parameters for the Complete-Cases Sample

	t0 (Baseline)		t1 (6 weeks)		t2 (post)		t3 (3-month FU)	
	B4DT (*n* = 60)	iCBT (*n* = 58)	B4DT (*n* = 59)	iCBT (*n* = 50)	B4DT (*n* = 58)	iCBT (*n* = 47)	B4DT (*n* = 57)	iCBT (*n* = 44)
Y-BOCS (primary)	26.13 (4.66)	27.25 (6.45)	25.86 (5.44)	25.14 (5.72)	14.36 (5.35)	22.23 (5.99)	14.75 (6.38)	20.39 (5.86)
OCI-R	28.43 (10.419	24.37 (11.29)	26.32 (10.61)	21.92 (11.54), *n* = 48	11.60 (7.30)	20.57 (11.40), *n* = 42	14.07 (7.68), *n* = 56	18.19 (9.87), *n* = 42
PHQ-9	9.30 (5.55)	9.19 (4.58),	8.05 (5.41)	7.36 (4.18), *n* = 47	4.33 (3.25)	6.79 (4.57), *n* = 42	5.39 (4.25), *n* = 56	7.02 (5.21), *n* = 41
WHOQOL	3.00 (0.92)	2.85 (0.94)	3.02 (0.82)	3.15 (0.72), *n* = 47	3.89 (0.67), *n* = 57	3.32 (0.76), *n* = 41	3.61 (0.76), *n* = 56	3.17 (0.89), *n* = 41
GAF	63.13 (9.01)	59.42 (10.38)	62.88 (9.26)	61.10 (9.71), *n* = 49	75.02 (9.30)	63.32 (8.61	76.32 (9.66)	67.77 (10.85)
OBQ-44	192.29 (46.53), n=59	170.25 (44.37)	189.64 (48.08)	155.64 (50.12), *n* = 47	127.53 (38.87), *n* = 57	144.63 (37.99), *n* = 40	127.63 (43.39), *n* = 56	143.68 (44.00), *n* = 40
RSE	29.28 (6.05)	28.64 (6.22)	29.27 (5.98)	29.38 (6.30), *n* = 47	33.04 (4.99), *n* = 57	30.90 (5.20), *n* = 41	33.02 (5.52), *n* = 56	31.10 (5.41), *n* = 41
BEAQ	51.43 (10.41)	50.27 (11.16)	50.20 (10.42)	48.66 (11.51), *n* = 47	38.81 (10.45), *n* = 57	46.34 (11.97), *n* = 41	38.45 (10.72), *n* = 56	45.12 (11.11), *n* = 40

Note. B4DT = Bergen 4-Day Treatment; BEAQ = Brief Experiential Avoidance Questionnaire; CI = confidence interval; FU = follow-up; GAF = Global Assessment of Functioning; iCBT = internet-based cognitive behavioral therapy; LSMD = least squares mean difference; OBQ-44 = Obsessive Beliefs Questionnaire–44; OCI-R = Obsessive-Compulsive Inventory–Revised; PHQ-9 = Patient Health Questionnaire–9; RSE = Rosenberg Self-Esteem Scale; WHOQOL = World Health Organization Quality of Life (single item); Y-BOCS = Yale-Brown Obsessive Compulsive Scale.

© 2026 Jelinek L et al. *JAMA Network Open*.

eTable 2. Complete-Case Sensitivity Analyses Using Repeated-Measures ANOVA and ANCOVA

	Repeated measures ANOVA (CC)		ANCOVA (CC)	
	Time	Time x Group	t0 to t2	t0 to t3 (primary analysis)
Y-BOCS (primary)	F(2.35, 230.11) = 145.19, P < .001, η_p^2 = .60	F (2.35, 230.11) = 32.09, P < .001, ηp^2 = .25	F(1, 102) = 68.68; P < .001; η_p^2 = .40)	F(1, 98) = 24.30; P < .001; η_p^2 = .20
OCI-R	F (2.32, 213.46) = 99.76, P < .001, η_p^2 = .52	F(2.32, 213.46) = 37.12, P < .001, η_p^2 = .287	F(1, 97) = 64.31, P < .001, η_p^2 = .40	F(1, 95) = 19.37, P < .001, η_p^2 = .17
PHQ-9	F(2.37, 213.57) = 20.17, P < .001, η_p^2 = .18	F(2.37, 213.57) = 5.77, P = .002, η_p^2 = .06	F(1, 97) = 13.61, P < .001, η_p^2 = .12	F(1, 94) = 5.00, P = .028, η_p^2 = .05;
WHOQOL	F(2.45, 220.74) = 21.76, P < .001, η_p^2 = .20	F(2.45, 220.74) = 8.36, P < .001, η_p^2 = .09	F(1, 95) = 19.37, P < .001, η_p^2 = .17	F(1, 94) = 7.49, P = .007, η_p^2 = .07.
GAF	F(2.53, 248.05) = 80.07; P < .001; η_p^2 = .45	F(2.53, 248.05) = 14.92; P < .001; η_p^2 = .13	F(1, 102) = 50.75, P < .001, η^2_p = .33	F(1, 98) = 16.25; P < .001; η_p^2 = .14
OBQ-44	F(2.04, 179.49) = 60.31, P < .001, η_p^2 = .41	F(2.04, 179.49) = 26.31, P < .001, η_p^2 = .23	F(1, 93) = 14.79, P < .001, η_p^2 = .14.	F(1, 92) = 14.10, P < .001, η_p^2 = .13
RSE	F(2.35, 211.39) = 22.13, P < .001, η_p^2 = .20	F(2.35, 211.39) = 6.56, P < .001, η_p^2 = .07	F(1, 95) = 9.14, P = .003, η_p^2 = .09	F(1, 94) = 5.11, P = .026, η_p^2 = .05.
BEAQ	F(2.12, 188.32) = 36.61, P < .001, η_p^2 = .29	F(2.12, 188.32) = 12.31, P < .001, η_p^2 = .12	F(1, 95) = 18.05, P < .001, η_p^2 = .16;	F(1, 93) = 14.12, P < .001, η_p^2= .13

Note. ANOVA = analysis of variance; ANCOVA = analysis of covariance; B4DT = Bergen 4-Day Treatment; BEAQ = Brief Experiential Avoidance Questionnaire; CC = complete cases; CI = confidence interval; FU = follow-up; GAF = Global Assessment of Functioning; iCBT = internet-based cognitive behavioral therapy; LSMD = least squares mean difference; OBQ-44 = Obsessive Beliefs Questionnaire–44; OCI-R = Obsessive-Compulsive Inventory–Revised; PHQ-9 = Patient Health Questionnaire–9; RSE = Rosenberg Self-Esteem Scale; WHOQOL = World Health Organization Quality of Life (single item); Y-BOCS = Yale-Brown Obsessive Compulsive Scale; t0 = baseline; t2 = posttreatment (8 weeks); t3 = 3-month follow-up.

© 2026 Jelinek L et al. *JAMA Network Open*.

eTable 3. Sensitivity Analysis Including Randomization Block as an Additional Fixed Effect: Baseline Means, Adjusted Outcomes, Standardized Effect Sizes, and Mixed Models for Repeated Measures (MMRM)

Outcome	Group	Baseline	t1	t2			t3			*p* (Group x Time)
		Mean (*SD*)	Mean (*SE*)	Mean (*SE*)	LSMD (95% CI)	Hedges' *g* (95% CI)	Mean (*SE*)	LSMD (95% CI)	Hedges' *g* (95% CI)	
Y-BOCS (primary)	B4DT	26.13 (4.63)	25.93 (0.52)	14.47 (0.63)	−7.88 (−9.74 to −6.03)	−1.64 (−2.03 to −1.26)	14.83 (0.75)	−5.61 (−7.85 to -3.37)	−0.98 (−1.37 to −0.59)	<.001
	iCBT	27.25 (6.42)	24.75 (0.57)	22.35 (0.69)			20.44 (0.84)			
OCI-R	B4DT	28.43 (10.35)	25.03 (0.77)	10.20 (0.92)	−11.56 (−14.36 to -8.75)	−1.65 (−2.04 to −1.25)	12.69 (0.97)	−7.39 (−10.33 to -4.46)	−1.01 (−1.41 to −0.61)	<.001
	iCBT	24.37 (11.22)	23.29 (0.86)	21.76 (1.06)			20.08 (1.11)			
PHQ-9	B4DT	9.30 (5.52)	7.90 (0.47)	4.12 (0.49)	−2.94 (−4.45 to −1.43)	−0.78 (−1.17 to −0.38)	5.27 (0.60)	−2.36 (−4.18 to −0.54)	−0.52 (−0.92 to −0.12)	.002
	iCBT	9.19 (4.55)	7.62 (0.53)	7.06 (0.58)			7.63 (0.69)			
WHOQOL	B4DT	3.00 (0.92)	3.02 (0.08)	3.89 (0.09)	0.58 (0.31 to 0.85)	0.86 (0.46 to 1.26)	3.62 (0.11)	0.49 (0.18 to 0.81)	0.62 (0.22 to 1.02)	<.001
	iCBT	2.85 (0.94)	3.16 (0.09)	3.32 (0.10)			3.13 (0.12)			

© 2026 Jelinek L et al. *JAMA Network Open*.

Outcome	Group	Baseline	t1	t2			t3			*p* (Group x Time)
		Mean (*SD*)	Mean (*SE*)	Mean (*SE*)	LSMD (95% CI)	Hedges' *g* (95% CI)	Mean (*SE*)	LSMD (95% CI)	Hedges' *g* (95% CI)	
GAF	B4DT	63.13 (8.96)	62.00 (0.74)	74.10 (0.97)	9.72 (6.83 to 12.61)	1.31 (0.92 to 1.70)	75.43 (1.15)	6.45 (2.98 to 9.92)	0.73 (0.34 to 1.13)	<.001
	iCBT	59.42 (10.32)	62.34 (0.82)	64.38 (1.08)			68.98 (1.31)			
OBQ-44	B4DT	192.29 (46.26)	181.28 (3.34)	116.39 (5.23)	−38.73 (−54.75 to −22.70)	−0.99 (−1.40 to −0.58)	116.68 (5.40)	−41.77 (−58.25 to −25.28)	−1.04 (−1.45 to −0.63)	<.001
	iCBT	170.25 (44.11)	166.73 (3.76)	155.11 (6.07)			158.45 (6.24)			
RSE	B4DT	29.28 (6.02)	29.22 (0.48)	33.13 (0.52)	2.46 (0.89 to 4.03)	0.62 (0.22 to 1.02)	33.18 (0.61)	2.34 (0.48 to 4.20)	0.50 (0.10 to 0.91)	.002
	iCBT	28.64 (6.19)	29.43 (0.55)	30.67 (0.60)			30.85 (0.71)			
BEAQ	B4DT	51.43 (10.35)	49.56 (0.94)	37.82 (1.35)	−9.49 (−13.58 to −5.41)	−0.93 (−1.32 to −0.53)	37.34 (1.43)	−9.29 (−13.64 to −4.94)	−0.85 (−1.26 to −0.45)	<.001
	iCBT	50.27 (11.10)	49.81 (1.07)	47.31 (1.55)			46.63 (1.65)			

Note. Data are presented as means (*SD*) at baseline (t0) and as estimated marginal means (*SE*) at t1, t2, and t3 derived from linear mixed models adjusted for baseline values and including randomization block as an additional fixed effect (post hoc sensitivity analysis). Least-squares mean differences (LSMD) represent adjusted differences between B4DT and iCBT (B4DT minus iCBT) at t2 and t3. Standardized mean differences (Hedges' *g*) were calculated by dividing the adjusted mean differences by the square root of the residual variance estimated from the mixed model at the corresponding follow-up assessment, applying a small-sample correction based on the Satterthwaite degrees of freedom. Confidence intervals for Hedges' *g* were obtained by applying the same transformation to the confidence intervals of the adjusted mean differences. *P* values correspond to the group × time interaction from the mixed model.

© 2026 Jelinek L et al. *JAMA Network Open*.

Abbreviations: B4DT = Bergen 4-Day Treatment; BEAQ = Brief Experiential Avoidance Questionnaire; CI = confidence interval; GAF = Global Assessment of Functioning; iCBT = internet-based cognitive behavioral therapy; *LSMD* = least squares mean difference; OBQ-44 = Obsessive Beliefs Questionnaire-44; OCI-R = Obsessive-Compulsive Inventory-Revised; PHQ-9 = Patient Health Questionnaire-9; RSE = Rosenberg Self-Esteem Scale; WHOQOL = World Health Organization Quality of Life (single item); Y-BOCS = Yale-Brown Obsessive Compulsive Scale; t0 = baseline; t1 = 6 weeks; t2 = posttreatment (8 weeks); t3 = 3-month follow-up.

© 2026 Jelinek L et al. *JAMA Network Open*.
