# Supplementary material for: Concentrated Exposure Therapy vs Internet-Based Cognitive Behavioral Therapy for Obsessive-Compulsive Disorder: A Randomized Clinical Trial
Source: JAMA Netw Open. 2026 Oct 19;9(10):e2639281. doi: 10.1001/jamanetworkopen.2026.39281 (PMC13681233; doi:10.1001/jamanetworkopen.2026.39281)
Supplement: Supplementary file 3 [file jamanetwopen-e2639281-s003.pdf]

Data Sharing Statement

Jelinek. Concentrated Exposure Therapy vs Internet-Based Cognitive Behavioral Therapy for Obsessive-Compulsive Disorder. *JAMA Netw Open*. Published October 19, 2026.
doi:10.1001/jamanetworkopen.2026.39281

Data
Additional Information: German Clinical Trials Register (DRKS, www.drks.de): #DRKS00034236 (07.05.2024)
Data available: Yes
Data types: Deidentified participant data
How to access data: Deidentified individual participant data are available from the corresponding author (E-mail: ljelinek@uke.de) upon reasonable request. Access is subject to applicable data protection regulations and may require a data use agreement and ethical approval.
When available: With publication

Supporting Documents
Document types: None

Additional Information
Who can access the data: researchers whose proposed use of the data has been approved
Types of analyses: for scientific purpose
Mechanisms of data availability: after approval of a proposal
